# Bipolar plasma vaporization using plasma-cutting and plasma-loop electrodes *versus* cold-knife transurethral incision for the treatment of posterior urethral stricture: a prospective, randomized study

**DOI:** 10.6061/clinics/2016(01)01

**Published:** 2016-01

**Authors:** Wansong Cai, Zhiyuan Chen, Liping Wen, Xiangxin Jiang, Xiuheng Liu

**Affiliations:** I Renmin Hospital of Wuhan University, Department of Urology, Wuhan, Hubei, China; II Renmin Hospital of Fuyang, Department of Urology, Hangzhou, Zhejiang, China

**Keywords:** Bipolar Plasma Vaporization, Cold-Knife Transurethral Incision, Plasma-Cutting Electrodes, Plasma-Loop Electrodes, Posterior Urethral Stricture

## Abstract

**OBJECTIVE:**

Evaluate the efficiency and safety of bipolar plasma vaporization using plasma-cutting and plasma-loop electrodes for the treatment of posterior urethral stricture. Compare the outcomes following bipolar plasma vaporization with conventional cold-knife urethrotomy.

**METHODS:**

A randomized trial was performed to compare patient outcomes from the bipolar and cold-knife groups. All patients were assessed at 6 and 12 months postoperatively via urethrography and uroflowmetry. At the end of the first postoperative year, ureteroscopy was performed to evaluate the efficacy of the procedure. The mean follow-up time was 13.9 months (range: 12 to 21 months). If re-stenosis was not identified by both urethrography and ureteroscopy, the procedure was considered “successful”.

**RESULTS:**

Fifty-three male patients with posterior urethral strictures were selected and randomly divided into two groups: bipolar group (n=27) or cold-knife group (n=26). Patients in the bipolar group experienced a shorter operative time compared to the cold-knife group (23.45±7.64 hours *vs* 33.45±5.45 hours, respectively). The 12-month postoperative Qmax was faster in the bipolar group than in the cold-knife group (15.54±2.78 ml/sec *vs* 18.25±2.12 ml/sec, respectively). In the bipolar group, the recurrence-free rate was 81.5% at a mean follow-up time of 13.9 months. In the cold-knife group, the recurrence-free rate was 53.8%.

**CONCLUSIONS:**

The application of bipolar plasma-cutting and plasma-loop electrodes for the management of urethral stricture disease is a safe and reliable method that minimizes the morbidity of urethral stricture resection. The advantages include a lower recurrence rate and shorter operative time compared to the cold-knife technique.

## INTRODUCTION

The operative methods for the treatment of posterior urethral stricture include endoscopic treatment and urethroplasty. Although urethroplasty has a high success rate, most urologists prefer endoscopic treatment because of its safety, simplicity and minimal invasiveness. Endoscopic treatment utilizes cold-knife and laser incision. Cold-knife urethrotomy is accompanied by a high recurrence rate (45% to 80%) 1-3. Various lasers have been tested to try to decrease the recurrence rate 3-7; however, due to the expensive cost of laser incision, it is not suitable for routine application. Some studies have confirmed that bipolar energy is an alternative energy source for the treatment of urethral strictures 8-12, and bipolar plasma cutting simultaneously incises and vaporizes the stricture, leading to decreased recurrent scar tissue formation 12. Therefore, we chose bipolar energy to treat posterior urethral strictures using a combination of plasma-cutting and plasma-loop electrodes.

In this study, we evaluated the efficacy and safety of endourethrotomy via bipolar energy using plasma-cutting and plasma-loop electrodes to treat posterior urethral strictures. Our study is the first randomized, prospective trial to compare patient outcomes following endourethrotomy performed with bipolar energy using a combination of plasma-cutting and plasma-loop electrodes or the cold-knife technique.

## MATERIALS AND METHODS

This study was approved by the Ethics Review Board of Wuhan University and written informed consent was obtained from each patient. From September 2008 to October 2013, 53 male patients with posterior urethral strictures were randomized into two groups: bipolar (n=27) or cold-knife group (n=26). All patients provided written informed consent, were blinded to the treatment, they received and were randomized using a “random number table”. The most commonly reported cause of stricture is trauma (83.7%) followed by iatrogenic causes (17.3%) 13. In our study, the most common causes of stricture were trauma (79.6%) and iatrogenic causes (20.4%). The following parameters were evaluated preoperatively for all patients: previous medical history and the results of physical examinations, serum biochemical analysis, urine analysis and culture, uroflowmetry and urethrography. The length of the stricture was measured by both ureteroscopy and uroradiography. The strictures were localized to the posterior urethra, which was directly visualized. In our study, the most frequent urethral stricture was membranous urethral stricture (85.6%) followed by prostatic urethral stricture (14.4%). In addition, operation time, hospitalization time and complications were recorded for all patients.

All urinary infections were cured prior to operation. All patients received a routine prophylactic antibiotic treatment of 1 g intravenous ceftriaxone 30 minutes before surgery and 1 g oral cefuroxime twice daily for 7 days after surgery.

Exclusion criteria: stricture length greater than 20 mm, multiple strictures (more than one site), more than one recurrence and loss to follow-up. All procedures were performed under general anesthesia.

**Figure 1:**
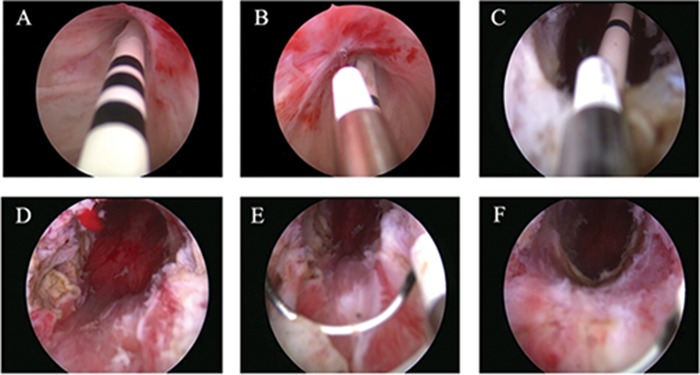
Treatment of posterior urethral stricture using bipolar energy administered via a combination of plasma-cutting and plasma-loop electrodes. (A) A 5F ureteral catheter was passed through the stricture. (B) A plasma-cutting electrode was inserted and vaporization was performed. (C) The passage was gradually broadened until a 24F cystoscope could pass through the urethra. (D) A plasma-cutting electrode was used for the urethra, which is generally not smooth. (E) A plasma-loop electrode was used. (F) The urethral passage became smoother.

The procedure was performed using a 19F cystoscope and plasmaKinetic^TM^</emph> cystoscope instruments (Gyrus plasmaKineticTM System, Gyrus Medical Ltd, Cardiff, UK). A 5F ureteral catheter was passed through the posterior urethral stricture (Figure 1A). The plasma-cutting electrode was inserted and the posterior urethral stricture was vaporized at 0, 120 and 240 degrees (Figure 1B). The passage was broadened gradually until the 24F cystoscope could pass through the stricture (Figure 1C). After vaporization of the urethral structures using the plasma-cutting electrode, the urethra was generally not smooth (Figure 1D). Then, the plasma-loop electrode (Figure 1E) was used for vaporization and the urethral passage became smoother (Figure 1F). An 18F silicone catheter remained in the urethra for 7 days.

For patients who were randomized to the cold-knife group, the urethral stricture scar tissue was incised at 0 degrees using a cold-knife; and an 18F silicone catheter remained in the urethra for 7 days.

Uroflowmetry was performed one month after the operation and the procedure was repeated every 6 months. After 12 months, all patients were evaluated by ureteroscopy to assess surgical outcomes.

During follow-up, the patient outcomes were considered successful if the maximum flow was >15 ml/sec without any obstructive symptoms and no recurrent strictures or an insignificant recurrent stricture was found by urethrography or ureteroscopy. If any intervention was recommended after the initial treatment due to re-stenosis, the surgery failed 14.

The statistical analyses were performed using SPSS 11.0 software (SPSS, USA). Categorical variables, such as recurrence rates and recurrence-free rates, were analyzed using Fisher's exact test. Numerical variables, such as Qmax values, stricture length and operative time were assessed using the Mann-Whitney U test. Differences with *p*-values<0.05 were considered significant.

## RESULTS

Twenty-seven male patients underwent bipolar plasma vaporization (bipolar group) and twenty-six male patients underwent cold-knife urethrotomy (cold-knife group). No patient was lost to follow-up. The procedures were successfully performed in all patients.

The mean patient age, preoperative stricture length and preoperative Qmax were similar between the two groups. There were no significant differences between the bipolar and cold-knife groups (Table 1).

No severe intra-or postoperative complications, such as false route, hemorrhage, epididymitis, bacteremia, erectile dysfunction or urinary incontinence, occurred in either group. The operative time was shorter in the bipolar group compared to the cold-knife group (23.45±7.64 h *vs* 33.45±5.45 h, respectively) (Table 2, *p*<0.001). Hospitalization time and the 1- and 6-month postoperative Qmax values were not significantly different between the two groups (Table 2, *p*>0.05). However, the 12-month postoperative Qmax was faster in the bipolar group compared to the cold-knife group (15.54±2.78 ml/sec *vs* 18.25±2.12 ml/sec, respectively) (Table 2, *p*<0.05). The recurrence-free rate was 81.5% (22/27) in the bipolar group and 53.8% in the cold-knife group (14/26). The recurrence rate was significantly lower in the bipolar group than the cold-knife group (Table 2, *p*<0.05).

## DISCUSSION

Cold-knife urethrotomy is the standard endoscopic procedure for the treatment of posterior urethral strictures. This operation is easy to perform but has a high recurrence rate 15. The reported recurrence rates for cold-knife urethrotomy range from 45% to 80% 1-3, which prompted us to search for a better therapeutic alternative. Various laser instruments have been investigated with the goal of decreasing the recurrence rate of posterior urethral stricture following endoscopic treatment 3-7. Recent studies have shown promising results with contact Nd:YAG and Ho:YAG lasers with success rates ranging from 80% to 93% 16,17. However, laser urethrotomy is not routinely used because of its high cost. Endoscopic urethrotomy using bipolar energy may be a safe, inexpensive and reliable procedure with good outcomes 8-12. However, the former studies utilized plasma-cutting electrodes without the assistance of the plasma-loop electrode. Thus, the scar tissue was difficult to remove. We used bipolar energy to treat posterior urethral strictures using a combination of plasma-cutting and plasma-loop electrodes.

Bipolar energy acts as follows: when high-frequency energy passes through a sodium chloride solution, a thin layer of vapor plasma is formed 18. The plasma contains energy-charged particles and causes disintegration through molecular dissociation 8,19. The temperature generated in this procedure is low and helps to restrict tissue damage to 1 mm. The depth of penetration of the Ho:YAG laser is 0.5 mm 16.

The cold-knife only incises the scar tissue, while bipolar energy incises and evaporates the scar tissue 20,21. Thus, bipolar energy decreases the recurrence of urethral scar tissue 22. Bipolar energy allows for simultaneous incision and vaporization of the stricture, making it easier to incise scar tissues. The cold-knife becomes increasingly blunt with use, especially when the knife is repeatedly used to incise hard scar tissue. This difference may explain why the operative time was shorter in the bipolar group than in the cold-knife group in our study. Basok et al. 10 used plasma energy for the treatment of urethral strictures and bladder neck contractures. The mean follow-up time was 13.8 months and the success rate was 77.3%. Based on that study, bipolar energy vaporization is a safe, reliable and inexpensive procedure with good results, negligible blood loss and minimal surgical morbidity. Therefore, bipolar energy vaporization can be considered a new therapeutic option for the endoscopic treatment of urethral strictures 10. Moldoveanu et al. 23 used a plasma-button electrode to treat secondary bladder neck sclerosis. In this report, the bipolar plasma vaporization technique was a promising modality for efficiently ablating obstructing fibrous tissue in patients with secondary bladder neck sclerosis 23. Geavlete et al. 12 performed a prospective, randomized trial to assess the efficiency and safety of bipolar plasma vaporization compared to monopolar transurethral resection for patients with bladder neck sclerosis. Geavlete et al. concluded that bipolar plasma vaporization is a valuable endoscopic treatment approach for bladder neck sclerosis. Moldoveanu et al. 24 compared the efficacy, safety and durability of bipolar plasma vaporization, monopolar transurethral resection 3 and cold-knife transurethral incision among patients with bladder neck sclerosis. The authors confirmed that plasma vaporization was successful in terms of postoperative recovery, therapeutic durability, surgical safety and urodynamic and symptom score parameters 24.

Our study confirmed that bipolar plasma vaporization using a combination of plasma-cutting and plasma-loop electrodes yielded a shorter mean operative time and higher recurrence-free rate than conventional cold-knife urethrotomy. Our study is the first to report the clinical experience of bipolar energy administered via a combination of plasma-cutting and plasma-loop electrodes for the treatment of posterior urethral strictures. The posterior urethral stricture cure rate was higher with bipolar energy vaporization than cold-knife urethrotomy. The bipolar energy method used here resulted in better patient outcomes than the cold-knife method and was as effective as laser treatment. Compared to conventional cold-knife urethrotomy, the significant advantages of our procedure were remarkable visibility, a smooth wound surface and less bleeding.

Considering the increasing bacterial resistance to antibiotics, mainly to ciprofloxacin, it is very important to administer appropriate prophylactic antibiotics. Kashanian et al. 25 reported a 24% resistance prevalence for ciprofloxacin after evaluating 10,000 E coli urine cultures. Das Gupta R et al. reported a ciprofloxacin resistance rate of 20%. The resistance to ceftriaxone was lower than ciprofloxacin 26. Therefore, we chose a sensitive antibiotic to control infection preoperatively based on the urine culture results. If the urine culture results were negative, we administered a 1 g dose of ceftriaxone 30 minutes before the surgery and 1 g doses of oral cefuroxime twice daily after the surgery. The appropriate duration of postoperative urethral catheterization is still controversial 27. In the literature, the reported duration of postoperative urethral catheterization ranges from 24 hours to 6 weeks 16. Prolonged catheterization is one of the most important risk factor for recurrence 28. Our protocol suggested postoperative urethral catheterization for 7 days. Furthermore, the catheter diameter should be appropriate. We chose an 18F silicone catheter. In our study, there were no severe urinary tract infections, such as epididymitis or bacteremia, in either group.

In our study, bipolar energy using a combination of plasma-cutting and plasma-loop electrodes is a safe, reliable method for the management of urethral stricture disease. Bipolar energy vaporization also yields a lower recurrence rate and shorter operative time compared with the cold-knife technique.

## AUTHORS CONTRIBUTION

Cai W conceived and designed the study. Chen Z performed the experiments. Wen L interpreted the data. Jiang X obtained the materials and reagents and helped drafting the manuscript. Liu X drafted the manuscript and revised it critically for important intellectual content.

## Figures and Tables

**Table 1:** Patient characteristics and preoperative values of the two groups.

Parameters	Bipolar group (n=27)	Cold-knife group (n=26)	*p* value
Age (years)	62.85±6.87	59.65±12.54	>0.05
Length of stricture (25)	12.23±2.48	11.45±2.89	>0.05
Preoperative Qmax (ml/sec)	4.65±1.88	4.72±1.57	>0.05

**Table 2:** Perioperative and follow-up data.

Parameters	Bipolar group (n=27)	Cold-knife group (n =26)	*p* value
Operative time (hours)	23.45±7.64	33.45±5.45	<0.01
Hospitalization time (days)	4.68±1.78	4.74±1.58	>0.05
1-month postoperative Qmax (ml/sec)	19.54±1.78	17.54±2.36	>0.05
6-month postoperative Qmax (ml/sec)	18.32±2.78	17.55±1.15	>0.05
12-month postoperative Qmax (ml/sec)	18.25±2.12	15.54±2.78	<0.05
Recurrence-free rate (%)	81.5% (22/27)	53.8% (14/26)	<0.05
Severe complications	0	0	
